# First Large-Scale DNA Barcoding Assessment of Reptiles in the Biodiversity Hotspot of Madagascar, Based on Newly Designed COI Primers

**DOI:** 10.1371/journal.pone.0034506

**Published:** 2012-03-30

**Authors:** Zoltán T. Nagy, Gontran Sonet, Frank Glaw, Miguel Vences

**Affiliations:** 1 Joint Experimental Molecular Unit, Royal Belgian Institute of Natural Sciences, Brussels, Belgium; 2 Zoologische Staatssammlung München, München, Germany; 3 Zoological Institute, Technical University of Braunschweig, Braunschweig, Germany; Centre National de la Recherche Scientifique, France

## Abstract

**Background:**

DNA barcoding of non-avian reptiles based on the cytochrome oxidase subunit I (COI) gene is still in a very early stage, mainly due to technical problems. Using a newly developed set of reptile-specific primers for COI we present the first comprehensive study targeting the entire reptile fauna of the fourth-largest island in the world, the biodiversity hotspot of Madagascar.

**Methodology/Principal Findings:**

Representatives of the majority of Madagascan non-avian reptile species (including Squamata and Testudines) were sampled and successfully DNA barcoded. The new primer pair achieved a constantly high success rate (72.7–100%) for most squamates. More than 250 species of reptiles (out of the 393 described ones; representing around 64% of the known diversity of species) were barcoded. The average interspecific genetic distance within families ranged from a low of 13.4% in the Boidae to a high of 29.8% in the Gekkonidae. Using the average genetic divergence between sister species as a threshold, 41–48 new candidate (undescribed) species were identified. Simulations were used to evaluate the performance of DNA barcoding as a function of completeness of taxon sampling and fragment length. Compared with available multi-gene phylogenies, DNA barcoding correctly assigned most samples to species, genus and family with high confidence and the analysis of fewer taxa resulted in an increased number of well supported lineages. Shorter marker-lengths generally decreased the number of well supported nodes, but even mini-barcodes of 100 bp correctly assigned many samples to genus and family.

**Conclusions/Significance:**

The new protocols might help to promote DNA barcoding of reptiles and the established library of reference DNA barcodes will facilitate the molecular identification of Madagascan reptiles. Our results might be useful to easily recognize undescribed diversity (i.e. novel taxa), to resolve taxonomic problems, and to monitor the international pet trade without specialized expert knowledge.

## Introduction

The elementary question how many species of eukaryotic organisms live on Earth has in the past decades led to remarkable controversies [1]–[5]. Current estimates of global species numbers differ enormously, ranging from 2 to 100 million, while ca. 1.7–1.9 million species have thus far been formally described. However, there is a broad agreement that the highest proportion of Earth's biodiversity—expressed in species numbers—is yet to be discovered and described. Even in several of the most prominent animal groups such as vertebrates and butterflies, a remarkable proportion of undescribed diversity is being discovered (e.g. Lepidoptera: [6]; fish: [7]; amphibians: [8]–[10]). Given that just 16,000–20,000 species are being described per year [11], [12] and traditional taxonomy involves high costs (estimated between $39,000–$122,000/species including salaries etc. [13]), strategies are required to speed up both the process of species discovery and species delimitation and description. A solution for the second of these challenges, the Linnean shortfall [14], is not in sight. Besides more posts for professional taxonomists [15], fundamentally new ideas and approaches will be required, especially in hyperdiverse groups such as small insects or nematodes. On the contrary, the initial identification of new species has been much facilitated and accelerated by DNA-based methods, and the term DNA barcoding has been proposed [16]–[19]. Similar to a long standing practice in microbiology [20], a short DNA sequence of a standard marker is used for species identification—in animals typically the mitochondrial gene for cytochrome oxidase subunit I (COI). This molecular survey method has been applied in a number of vertebrate taxa (e.g. birds: [21], [22]; fish: [23]) and invertebrates (e.g. spiders: [24], Lepidoptera: [25]–[27], marine invertebrates: [28], and Heteroptera: [29]). DNA barcoding has the potential to increase the rate of discovery enormously [30] and to discover unexpected genetic diversity such as in butterflies [31]–[32] or in amphipods [33], [34]. From practical point of view, DNA barcoding requires a comprehensive reference database [35]. Such reference data sets are being assembled by the barcoding campaigns initiated by the Consortium for the Barcode of Life (CBOL). For vertebrates, campaigns focusing on fish, birds and mammals have been started: the ‘Fish Barcode of Life Initiative’ (FISH-BOL, www.fishbol.org; [36]), the ‘Shark Barcode of Life’ project (www.sharkbol.org), the ‘All Birds Barcoding Initiative’ (ABBI, www.barcodingbirds.org) and the ‘Mammal Barcode of Life’ (www.mammaliabol.org) project. A new barcoding campaign called ColdCode dedicated to amphibians and non-avian reptiles has been announced in September 2011, and new COI primers for amphibians have been published [37]. Although molecular data are regularly used to discover and delimit new species of reptiles, no large-scale DNA barcoding effort has so far targeted an entire species-rich reptile fauna of a large region.

To facilitate reading, we will in the following use the traditional term ‘reptiles’ for species included in the vertebrate orders Squamata, Testudines, Crocodylia, and Rhynchocephalia, i.e. Sauropsida excluding birds. We continue using the term ‘reptiles’ for practical reasons only and without any phylogenetic relevance.

Due to technical problems in the amplification and evaluation of COI sequences in amphibians and reptiles linked to a high variability of sequences including priming sites, authors of previous attempts [38] have preferred the use of a fragment of the mitochondrial 16 S rRNA gene for DNA barcoding (*sensu lato*) despite its non-coding characteristics and resulting alignment problems. Although recent publications claim that the difficulties of COI amplification in amphibians can be overcome [39], various teams have experienced problems. Therefore, COI as marker for molecular identification and for phylogenetic and phylogeographic studies has been rarely used by herpetologists. Consequently, there is a serious lack of DNA barcodes for amphibians and reptiles, and according to the Barcode of Life Data Systems [18] DNA barcodes of most taxa of reptiles (even on higher taxonomic levels) inhabiting Madagascar are virtually absent. This highlights the need for development and testing of primers and amplification strategies for these organisms [40].

Here we start filling this gap and provide a DNA barcoding assessment of the reptile fauna of Madagascar, the fourth-largest island in the world that has been flagged as one of the most important hotspots for biodiversity conservation [41] and as a model region to study species diversification [42]. Madagascar's biota is most fascinating due to the unique level of endemism associated with high alpha diversity [43]. Approximately 92% of the non-marine species of Madagascan reptiles (i.e. excluding sea turtles and sea snakes) are endemic to the island, and many of them are furthermore microendemic to very small ranges [42]. By 2007, around 370 nominal species of reptiles were known from Madagascar [44]. Thanks to intensive and integrative taxonomic work in the last approximately 20 years, this number is continuously growing. Since 2007 alone, 22 new species have been described, bringing the total number of nominal species of reptiles from Madagascar up to 393 at present. Genetic, phylogenetic and phylogeographic information on several taxonomic groups is accumulating as well [43], [45]. At the same time, increased and insufficiently controlled human activities affecting the habitats seriously threaten the long-term sustainment of this fauna and make a comprehensive assessment of the diversity a high priority.

In this study we aim to characterize the majority of Madagascan reptiles by DNA barcoding based on a newly developed set of reptile-specific primers for COI. We compare the performance of this method to assess the species diversity of a large biodiversity hotspot, exemplified by the Madagascan “microcontinent” with its clades of reptiles of different temporal diversification background, and test the performance of the method depending on barcode length and completeness of taxon sampling.

## Materials and Methods

### Ethics statement

No experiments were conducted using living animals. Furthermore, none of the samples were specifically collected for this project. We exclusively used museum samples which were already available and were deposited in a tissue bank.

All field researches and collecting of specimens were approved by the Madagascan Ministère de l'Environnement, des Eaux et des Forêts (Direction des Eaux et Forêts, DEF) under the following permits: 156-MEF/SG/DGEF/DADF/SCB dated 12 December 2002; 238-MINENVEF/SG/DGEF/DPB/SCBLF dated 14 November 2003; 238-MINENV.EF/SG/DGEF/DPB/SCBLF/RECH dated 22 December 2004; 272-MINENV.EF/SG/DGEF/DPB/SCBLF/RECH dated 8 November 2005; 298-MINENV.EF/SG/DGEF/DPB/SCBLF/RECH dated 22 December 2006; 036/08 MEEFT/SG/DGEF/DSAP/SSE dated 30 January 2008; 26/09/MEEFT/SG/DGEF/DSAP/SLRSE dated 3 February 2009; 48/09/MEEFT/SG/DGEF/DSAP/SSE dated 9 March 2009; 188/09/MEEFT/SG/DGEF/DSAP/SSE dated 16 September 2009; 195/09/MEEFT/SG/DGEF/DSAP/SSE dated 28 September 2009. Export of specimens was approved by the DEF under permits: 063C-EA02/MG03, dated 26 February 2003; 094C-EA03/MG04, dated 1 March 2004; 103C-EA03/MG05, dated 15 March 2005; E 1400/06, dated 1 June 2006; 055N-EA03/MG10, dated 25 March 2010. Import of species protected by CITES into Germany was approved by the German authorities (Bundesamt für Naturschutz). Voucher specimens were euthanized using proved methods (e.g. anaesthesia with ketamine, followed by ketamine overdosis) that do not require approval by an ethics committee.

### Sampling

We sampled 468 specimens of Madagascan reptiles mostly deposited in publically accessible natural history collections (for list of samples, see Table S1). About 420 of these samples were determined to belonged to 251 nominal species. Another ca. 50 samples could not be reliably assigned to any nominal species based on morphology and had high sequence divergences to the other samples included (candidate species as defined below). Samples belong to nine squamate families, Chamaeleonidae, Iguanidae (Opluridae), Gerrhosauridae, Scincidae, Gekkonidae, Boidae, Lamprophiidae, Psammophiidae (the latter two snake families were formerly included in Colubridae *sensu lato*), Typhlopidae (*sensu lato*, i.e. also including the recently described Xenotyphlopidae), and to the tortoise and turtle families Testudinidae and Pelomedusidae. Due to practical problems such as restricted distribution and rareness of species, identification problems, or inclusion of species in CITES, only a limited amount of samples were available for the majority of species (on average, 1.7 samples per species, ranging from 1 to 5). Therefore, our sampling strategy was to include only single samples for most species but to select these as carefully as possible, making sure their identification is correct and they are backed by a traceable voucher specimen. For this purpose we chose, whenever possible, samples from type material (holo- or paratypes) or collected at or near the type locality. All collecting localities are listed in Table S1. Altogether about 85 species were represented by at least one specimen from the type locality or its surroundings, and altogether 23 species were represented by sequences from type specimens (marked in Table S1).

### Lab methods

Specimens and samples were collected from numerous localities in Madagascar in the years 2000–2009. Tissue samples removed from freshly collected specimens were stored in 95–99% ethanol. Total genomic DNA was extracted with commercial kits, we used the NucleoSpin Tissue Kit (Macherey-Nagel, Germany) and the Qiagen DNeasy 96 Blood & Tissue Kit (Qiagen, Germany).

We newly designed a degenerative primer pair based on squamate mitochondrial genome sequences available in GenBank. This primer pair amplifies the standard barcoding region (maximal length: 664 bp) of the cytochrome oxidase I gene [46]: RepCOI-F: 5′-TNT TMT CAA CNA ACC ACA AAG A-3′ and RepCOI-R: 5′-ACT TCT GGR TGK CCA AAR AAT CA-3′. The PCR protocol followed the profile of 94°C for 3 min; 40 cycles of 94°C for 40 s, 48.5°C for 30 s and 72°C for 60 s; 72°C for 7 min, and subsequent storage at 4°C. PCR products were visualized on ca. 1.2% agarose gel, and purified using the NucleoFast 96 PCR Plate (Macherey-Nagel, Germany). PCR products were sequenced bidirectionally using the same primers. The sequencing was mainly carried out on an ABI 3130xl automated capillary sequencer using BigDye v1.1 chemistry and following the manufacturer's instructions (Life Technologies, USA). Some samples were resolved on automated sequencers by a commercial service provider for DNA sequencing.

### Evaluation

Sequences were assembled, aligned and checked for their quality using the SeqScape v2.5 software (Life Technologies, USA). Sequences of doubtful mitochondrial origin (e.g. sequences showing suspiciously high divergence to any other COI sequence or where internal stop codons were detected), were removed from the final data set. We only used sequences with a length encompassing at least 90% of the standard animal barcoding region (i.e. at least 600 bp) as a high-quality read and based our calculation on success rates on this yardstick. The alignment was submitted to a test of substitution saturation [47], [48] as implemented in DAMBE v5.2.34 [49]. In addition, transitions and transversions were plotted against Kimura 2-parameter (K2p) divergences to visualize possible saturation at higher divergence level. Neighbor-joining (NJ) trees based on K2p distances were calculated using MEGA5 [50]. We also used Bayesian inference and calculated a Bayesian consensus tree and posterior probabilities supporting nodes using MrBayes v3.1.2 [51]. For the latter analysis, the best-fit nucleotide substitution model was selected by jModeltest [52] using AIC(c) and BIC. In the Bayesian analysis, two parallel runs with four chains each were run for 10 million generations. The first 60% of the trees were discarded, the convergence of the chains was monitored by Tracer v1.5 [53]. A maximum likelihood (ML) tree was calculated and a ML bootstrap test with 100 replicates was performed using the DIVEIN web server [54] that is based on PhyML v3.0 [55].

To assess the number of deep genealogical lineages in our dataset that represent potentially undescribed species, we first determined a threshold value of genetic divergences that typically are found among closely related species. For this we used a reference subset of our data with sequences of unambiguously identified specimens. This reference set included 251 well-delimited nominal species represented by single specimens and belonging to eight squamate families. Psammophiid snakes, turtles and tortoises were excluded from these analyses due to the low number of specimens and species included. Average interspecific genetic divergence (both K2p and p distance) was calculated in each squamate family involved. In addition, average values of genetic distance were calculated among all well-supported sister species (supported by >70% bootstrap and selected based on the initial NJ analysis) in each of those eight families. These average divergences were then used as family-specific thresholds for candidate species (as defined according to Vieites et al. [8]) in the complete data set. We counted as candidate species those unidentified lineages differentiated by a genetic divergence above the family-specific threshold from their closest relative.

We assessed by simulations the effect of a less comprehensive taxon sampling (only a certain proportion of species included) and of a reduced genetic sampling (shorter DNA fragment used) on the results. In the first test series (taxon subsampling), four times 100 random subsets of our data set were created, including 75, 50, 25, and 10% of the species included in the original data set. In the second test series, we aimed to evaluate the performance of the “mini-barcode” approach [56]. Fragments stretching over the first 100, 200, 300, and 450 bp of the barcoding region were selected, respectively. For each subsets produced in both test series (i.e. 400 subsets in the subsampling with variable number of taxa, 4 in the subsampling with different fragment size), NJ trees based on K2p distances were calculated, and bootstrap analyses with 1000 replicates were carried out. The topology and bootstrap values of these trees were then compared with the most complete NJ tree obtained with the original data set. We checked in all trees whether genera and families were clustered as groups and recorded the support values of those groups. Groups with single specimens were obviously excluded from the analysis.

One goal of DNA barcoding is to match an unidentified sample to a certain species by comparison with existing sequences of well-identified species. When a species-level identification is ambiguous because of an incomplete set of references, it often is desirable to obtain at least a reliable attribution of the non-identified sample to a higher taxon, for example a genus. In incomplete databases, identification tends to decrease with increasing taxon coverage [57] but obviously with a complete set of reference sequences of all species, identification success will be very high. We tested the success of COI sequences to correctly cluster with a sequence to a higher clade by selecting a number of genera reliably known to be monophyletic from previous phylogenetic studies. (*Liopholidophis*, *Lygodactylus*, *Phelsuma*, *Trachylepis*, *Uroplatus* and *Zonosaurus*). We scored whether these genera became non-monophyletic in COI trees at lower taxon sampling or with shorter sequences, and whether this topology received high support values. Non-monophyly in this case implies a wrong genus-level assignment of at least some of the sequences and in a tree-based approach is thus an indicator to the reliability by which sequences of unknown identity without a clear match in a reference database can be attributed to genus-level. Tests were performed on the complete data set, on the ‘mini-barcode’ data set (all sequences, 100 bp) and on 100 subsets including 10% of the species (full sequence length). The automation of the subsampling and the analysis of NJ trees was implemented in an R script (Sonet & Nagy unpublished).

## Results

### DNA barcodes for Madagascan reptiles

Using the new primer pair, we produced COI sequences for the majority (ca. 64%) of the Madagascan species of reptiles. Beyond well recognizable and “established” species, we also included several confirmed or unconfirmed candidate species [8] and indicate these as ‘sp.’ or ‘aff.’.

The success rate of the PCR amplification and DNA sequencing was constantly high even when only degraded DNA or minimal amounts of tissue samples were available. Nevertheless, in 21 cases when sequencing failed in a first attempt, we re-extracted DNA from another tissue sample of the same specimen and repeated the downstream process with unchanged conditions (as an approximation to automated, high-throughput procedures). In several cases, repeated attempts of amplification or sequencing failed again. In total, 489 tissue samples were taken, representing 468 specimens. After quality checks, 396 sequences with a maximal length of 664 bp (range of sequence length: 604–664 bp) were used to build a data set for the analyses. This corresponded to an ultimate success rate of 84.6%. Success rate varied over the taxonomy, for example turtles and geckos failed more often than chameleons and snakes (Table 1). BoL standard primers were not extensively tested after initial failure.

**Table 1:** Success rates of DNA sequencing according to the taxonomy.

Family	No. of tissue samples (duplicates included)	No. of specimens represented	Successful	Failed	Success rate (%)
Gekkonidae	149	**139**	**101**	38	**72.7**
Lamprophiidae	111	**107**	**94**	13	**87.9**
Chamaeleonidae	97	**96**	**94**	2	**97.9**
Scincidae	67	**65**	**62**	3	**95.4**
Gerrhosauridae	21	**18**	**16**	2	**88.9**
Testudinidae	11	**10**	**2**	8	**20.0**
Iguanidae	10	**10**	**8**	2	**80.0**
Typhlopidae	8	**8**	**6**	2	**75.0**
Boidae	5	**5**	**5**	0	**100.0**
Psammophiidae	5	**5**	**5**	0	**100.0**
Pelomedusidae	5	**5**	**3**	2	**60.0**
TOTAL:	489	**468**	**396**	72	AVERAGE:**84.6**

The substitution saturation analysis showed little overall saturation, i.e., the index of substitution saturation, Iss, was always significantly lower than the critical value of the index of substitution saturation, Iss.c; [47], [48]. The graph showing transitions and transversions plotted against divergence (Figure S1), however, indicated saturation at higher divergence level.

**Figure 1:**
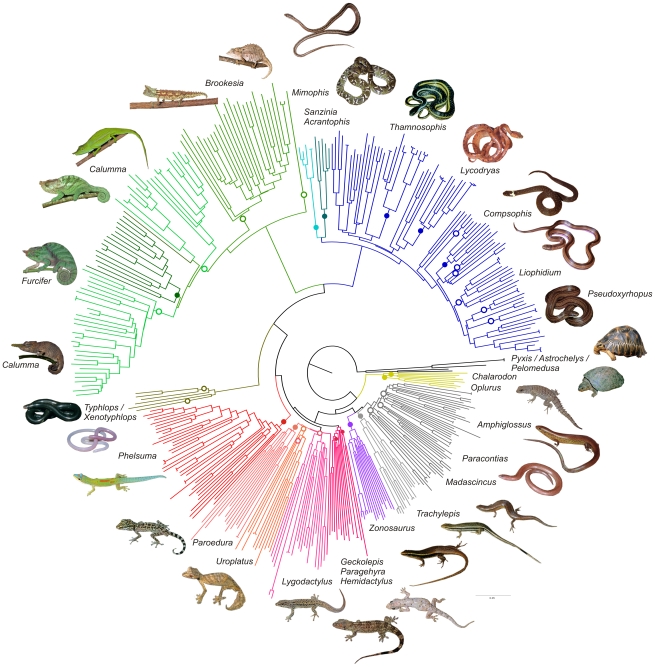
Neighbor-joining tree based on COI sequences of Madagascan reptiles. Inset photos and genus names refer to representative species-rich genera (for a tree with complete taxon names, see Supplementary Materials). Filled circles mark groups corresponding to these genera; when a genus was reconstructed paraphyletic, open circles denote the placement of its members (not shown in all cases due to graphical reasons).

The DNA barcoding approach generally worked well for most Madagascan reptiles as the Folmer fragment of the COI gene [46] distinguished well on specific as well as on higher taxonomic levels (Figure 1). All applied tree-reconstruction methods (i.e. neighbor-joining based on genetic distance, maximum likelihood and Bayesian inference) recovered many well supported groups corresponding to ranks of species, genera and even families. These tree-reconstruction analyses were largely concordant in topology (see Figures S2, S3 and S4 for NJ, Bayesian and ML trees, respectively) and, we relied on the NJ tree for further analyses.

### High sequence diversity on the generic and specific level

The depth of diversity calculated on average pairwise genetic distances between well-defined (‘good’) species varied remarkably among the investigated families. The lowest divergence was found among the Madagascan boas (Boidae). In general, snakes, gerrhosaurids and iguanas showed lower average divergence, while skinks, chameleons and especially geckos showed higher overall pairwise divergence (Table 2).

**Table 2:** Data on genetic divergences in various Madagascan reptile lineages, and approximate number of candidate species found in our data set (see details in text).

Family	No. of ‘good’ species served as reference	Average genetic divergence between ‘good’ species (K2p/p values in %)	No. of sister pairs	Average genetic divergence between sister pairs (K2p/p values in %) used as threshold	No. of identified ‘candidate species’
Boidae	3	13.4/11.9	1	6.6/6.2	**1**
Lamprophiidae	52	20.2/17.3	6	9.0/8.3	**10–11**
Typhlopidae	6	18.6/16.3	1	6.9/6.5	**0**
Iguanidae	6	18.5/16.1	2	14.9/13.3	**1**
Gekkonidae	70	29.8/24.2	7	15.2/13.3	**9–11**
Gerrhosauridae	13	19.2/16.7	1	13.9/12.5	**0**
Scincidae	42	22.2/18.9	5	6.5/6.1	**6–8**
Chamaeleonidae	59	23.7/19.9	7	10.0/9.1	**14–16**

The average divergences between species in sister species pairs were even more obviously different among clades, although usually a low number of sister pairs were found with high bootstrap support; snakes, skinks and chameleons showed comparatively low divergences between sister pairs on average, while iguanas, geckos and gerrhosaurids showed high divergences. Based on these latter values used as threshold, we counted the number of candidate species (as summarized in Table 2). In total, our analysis revealed 41–48 candidate species in our limited data set that represent 16–19% of the included more than 250 nominal species, and 10–12% of the known diversity of reptiles in Madagascar. We emphasize that these are minimum values directly inferred by counting above-threshold lineages in our data set. Estimating the total proportion and number of candidate species across Madagascan reptiles is at present not possible because too few species in our data set are represented by multiple sequences and too few geographic locations per species have been sampled.

A few examples involving several candidate species and particularly high divergences were as follows: the chameleons in the *Calumma nasutum* complex (11.3–18.9% K2p divergence), the geckos in the *Phelsuma lineata* complex (4.2–18.6%), the skink *Trachylepis gravenhorstii* (6.2–14.0%), and the snake *Pseudoxyrhopus tritaeniatus* (7.8–9.9%). In some cases, specimens thought to belong to the same species clustered paraphyletically in clearly independent, well supported groups (see Figures S2, S3, S4 for details). The most striking example was that of the terrestrial snake species *Liophidium torquatum* where two deeply divergent lineages were found. One of them was closely related to the single Comoran species *Liophidium mayottensis* that we included in the otherwise exclusively Madagascan data set. A few notable exceptions of no or very low genetic divergences between species were the *Phelsuma modesta* complex, the species pair *Phelsuma dubia* and *P. ravenala*, and the species *Brookesia antakarana* and *B. ambreensis*.

### The effect of taxon sampling

The complete data set included 57 genera of which 38 were represented by more than a single sequence. Twenty-two of these 38 genera clustered in monophyletic units, and 12 of them were supported by high bootstrap values (>90%) in the NJ tree. On the family level, nine of eleven families formed monophyletic groups, and eight of them were well supported in the NJ tree (Figure S2, Table S2).

**Figure 2:**
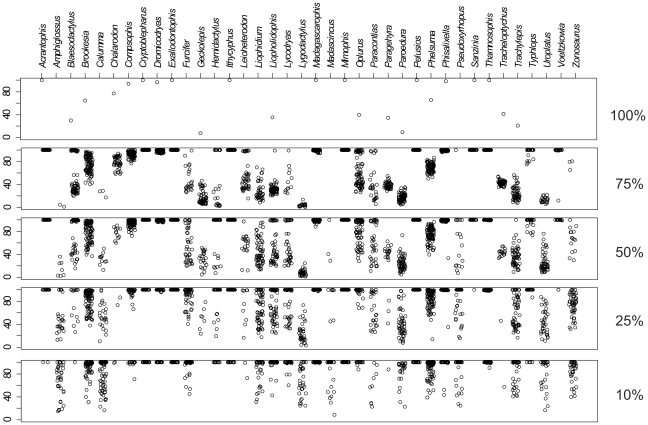
Plot of bootstrap values supporting monophyly of genera with different taxon sampling density. Values were obtained by neighbor-joining analyses including 100% (original data set), 75, 50, 25 and 10% (100 random subsets each) of the studied species, respectively. Support values for taxa recovered as non-monophyletic groups are not shown.

**Figure 3:**
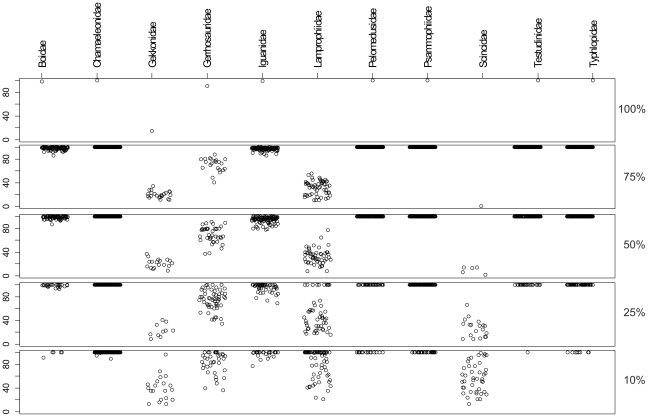
Plot of bootstrap values supporting monophyly of families with different taxon sampling density. Values were obtained by neighbor-joining analyses including 100% (original data set), 75, 50, 25 and 10% (100 random subsets each) of the studied species, respectively. Support values for taxa recovered as non-monophyletic groups are not shown.

Results of simulations with randomly selected subsets of 75, 50, 25, and 10% of the species were summarized in Table S2. Bootstrap values supporting genera (Figure 2) and families (Figure 3) ranged widely in the NJ trees obtained. However, on average, the number of strongly supported genera and families remained virtually unchanged in all analyses. Genera that were well supported in the original data set remained generally well supported at a lower taxon sampling. In contrast, genera and families that were weakly or not supported in the complete data set (i.e., showing bootstrap values <90%) sometimes became highly supported at lower taxon sampling depending on the set of species included in the simulation. This resulted in fluctuating bootstrap values between 0 and 100% (Figure 2, Table S2). Similar tendencies were observed on the family level (Figure 3). Seven families (Boidae, Iguanidae, Chamaeleonidae, Testudinidae, Pelomedusidae, Typhlopidae and Psammophiidae) were well supported irrespective of the depth of taxon sampling, while the other families investigated (i.e. Lamprophiidae, Scincidae, Gekkonidae and Gerrhosauridae) received increasing support with decreasing taxon sampling.

For the six species-rich genera known to be monophyletic, in 80% of the randomizations the monophyly was confirmed with high support values.

### The effect of marker length – barcodes versus mini-barcodes

**Figure 4:**
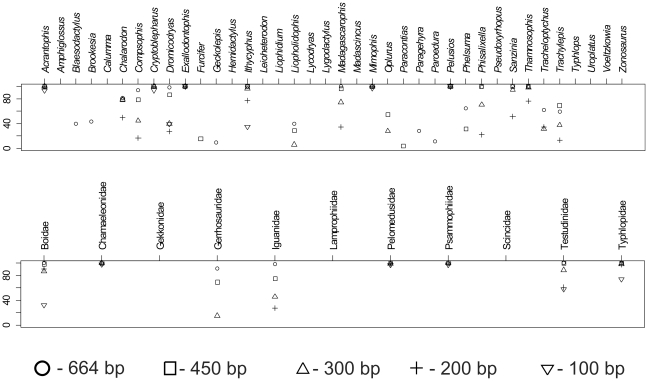
Plot of bootstrap values supporting genera and families with different DNA sequence length. Values for genera (top) and families (bottom) were obtained by neighbor-joining analyses based on DNA sequence fragments of 664 bp (original data set), 450, 300, 200 and 100 bp, respectively.

Neighbor-joining analyses based on shorter sequences (450, 300, 200, and 100 bp, respectively) showed that the number of well supported groups (with bootstrap values over 90%) decreased with shorter marker length (Figure 4), both on family- and genus-level. A few genera were found—mostly including a single or a couple of species only—which were highly supported in all subsets testing the effect of marker length. Also, none of the six species-rich monophyletic genera was supported significantly in the data set of mini-barcodes (100 bp). On the family level, even mini-barcodes of 100 bp length were sufficient to obtain highest support values in Chamaeleonidae, Pelomedusidae, and Psammophiidae, but low or no support was recovered for the other eight families. Results of distance-based comparisons showed that short marker sequences of 100 bp remained unique on species level, and, therefore, could be used for unambiguous identification of the samples.

## Discussion

### Promoting DNA barcoding of reptiles

In this study we propose a newly designed degenerated primer pair. It works well with several lineages of squamates at a constantly high success rate in concert with easy-to-use protocols (standard PCR procedure with a single annealing temperature). The main goal of our study is to establish a DNA barcode database for Madagascan reptiles and to test the utility of these COI sequences for identifying species and assigning them to major units (corresponding to genera and families). We do not specifically aim to screen Madagascar's reptile fauna for cryptic species, yet our data suggest or confirm a substantial proportion of undescribed diversity in this group, with over 40 candidate species newly identified. Our barcoding approach works universally, without conspicuous differences in PCR amplification or identification success recognizable among the main evolutionary lineages of squamates. A total of ca. 15% of the samples are not reliably amplified with this new primer pair, suggesting that multiplexing with several primers or designing clade-specific primers will be necessary in applications that rely on absolute success rates in such old and diverse groups as reptiles.

### The effect of sampling

The restricted amount of specimens per species included in our study permits only a limited assessment of intraspecific genetic variation. Hence, reliable comparisons of intraspecific *versus* interspecific diversity are difficult. In contrast, the high species coverage in our study is a suitable fundament to test the effect of missing taxa on the performance of DNA barcoding to correctly assign species to genera and other higher taxa, i.e., to provide a higher level taxonomic identification for sequences of unknown identity with no match in the database.

There is a long-standing debate on the effects of taxon (and character) sampling initiated in the 1990s, although focused to phylogenetic studies [58], [59].The overall agreement is that increased taxon sampling will typically result in higher phylogenetic accuracy (e.g. [58], [60]–[65] and many others). In particular, increased taxon sampling effects on reducing phylogenetic error (e.g. [54], [63], but other studies are contradictory [66]). The analysis of few taxa can be subject of strong biases, “which in turn produce high measures of repeatability (such as bootstrap proportions) in support of incorrect or misleading phylogenetic results” [67]. Moreover, more complex evolutionary models are mainly beneficial for larger taxon sampling. To test sampling effects and estimate expected errors, simulations (i.e. randomly selected trees) are necessary [64].

In our simulations, we opted for the neighbor-joining method based on distances due to its simplicity and high computational speed. Furthermore, NJ based on K2p distances is a commonly used clustering method in many DNA barcoding studies [17], [18], and also recommended as a ‘minimum’ standard method [68]. NJ yields results sufficiently similar to the results of likelihood- or parsimony-based phylogenetic analyses to be used as an approximation [69], especially with short DNA sequences where ML-based analyses may suffer from overparametrization.

Our simulations suggest two main trends: First, several taxonomic groups exist that always receive strong support irrespective of the depth of taxon sampling. These are mainly highly divergent evolutionary lineages. Second, we observe that the proportion of groups receiving high bootstrap support increases with decreasing taxon sampling. Apparently, the size of the unit does not determine the variation of support values in the different subsets. Anyway, some groups are not monophyletic in the most comprehensive (original) tree but become monophyletic in some simulations due to coincidental taxon sampling. This observation should be kept in mind for the evaluation of these gene-trees: high support values at low taxon sampling could potentially be misleading because they may not accurately reflect support for the entire group and are simply a consequence of missing data.

The effect of using mini-barcodes on the resolution of relationships is known [56]. Our observations show that the number of supported taxa (in this case genera and families) are lower when using shorter sequences but on the other hand even 100 bp sequences are able to assign many samples correctly to genus and family.

### COI-based clustering versus multigene phylogeny of Madagascan reptiles

DNA barcodes are usually not analyzed in a phylogenetic context. Especially at deeper divergences corresponding to higher taxonomic ranks, substitution saturation may become very substantial. Therefore, DNA barcodes may not be useful in phylogenetic reconstruction of higher taxa even with ‘maximal’ taxon sampling (e.g. Lepidoptera, [70]). Nevertheless, massively increased taxon sampling also increases phylogenetic signal in the data set [70], which can be exploited using other means. Besides this phylogenetic perspective, understanding whether sequences are correctly assigned to higher clades or not is also informative for barcoding because it indicates whether sequences of species not contained in the database will be correctly assigned to higher clades and taxa [57].

Although the primary goal of DNA barcoding is species identification and species discovery and not phylogenetic analysis, in many cases our results can be compared with multigene-based phylogenetic studies. Given that our barcoding data include numerous taxa that so far have remained phylogenetically unassessed, a number of tentative hypotheses can be drawn to be tested in future phylogenetic studies.

The COI tree supports the monophyly of many snake genera with maximum boostrap values in agreement with previous multigene phylogenies. The branching pattern of species within a given genus is remarkably to moderately congruent with that in the multilocus phylogenies of *Compsophis* [71], *Liophidium* [72], *Liopholidophis* [73], *Thamnosophis* [74], *Madagascarophis* [75], *Sanzinia* and *Acrantophis* [76], although a less complete sampling in several of the multigene trees does not allow exact comparisons. The polyphyly of the former snake genus *Stenophis*, only recently resolved by recognizing three monophyletic genera (*Lycodryas*, *Parastenophis* and *Phisalixella*; [77]) is also recovered, as is the polyphyly of the snake genus *Liopholidophis sensu lato*, splitted in the two monophyletic genera *Liopholidophis* and *Thamnosophis* [73]. The genus *Pseudoxyrhopus* appears to be polyphyletic as well. The main group includes *Heteroliodon fohy* (suggesting that this species is perhaps just a miniaturized member of *Pseudoxyrhopus*, [78]), while at least two other species (*P. heterurus* and *P.* cf. *imerinae*) form two independent lineages. Unfortunately, no comprehensive multilocus phylogeny is available for this complex.

Regarding chameleons, the polyphyly of the genus *Calumma* is in agreement with a previous study [79]. The genus *Furcifer* is recovered as monophyletic except for *F. balteatus* which is one of the most basal *Furcifer* species [79]. The dwarf chameleons of the genus *Brookesia* appear as monophyletic without *B. nasus* which together with *B. lolontany* forms the most basal *Brookesia* clade [80]. Among gerrhosaurids, *Tracheloptychus* is found to be nested within *Zonosaurus*, in agreement with a paraphyletic genus *Zonosaurus* [81].

The most taxonomically convoluted assemblage is that of the fossorial scincid lizards [82], [83]. Our COI-based tree shows the genera *Voeltzkowia*, *Pygomeles* and *Androngo* nested within *Amphiglossus*, a result consistent with the published phylogeny [82], implying that taxonomic changes are required. In the gecko genus *Paroedura*, several sister group relationships [84] are correctly recovered by COI sequences. Intrageneric relationships are also largely correctly inferred among the day-geckos of the genus *Phelsuma* [85]. For the leaf-tailed geckos (genus *Uroplatus*), we have a lower species coverage, and therefore a comparison with a phylogenetic study [86] is less informative. Our results on Madagascan iguanas – genera *Oplurus* and *Chalarodon* – confirm earlier findings [87].

Summarizing we conclude that our single-gene tree is remarkably informative in recovering shallow-level phylogenetic relationships (i.e. correct attribution to existing families, genera, and species groups in most cases). It also has the potential to point to possibly non-monophyletic species (e. g. *Phelsuma madagascariensis sensu lato*, *P. lineata sensu lato*) and genera (*Amphiglossus*, *Brookesia*, *Calumma*, *Zonosaurus*), which are so far not resolved by morphological data sets. However, in cases when results seem to be in conflict with current taxonomy, we suggest a taxonomic re-assessment to confirm or redefine species boundaries. This should be made by in-depth investigations using additional molecular markers and morphological traits.

### Utility of DNA barcodes of Madagascan reptiles

Similar to Madagascan frogs [8], taxonomy of squamates is limping far behind the collection of specimens of candidate species by intensive field surveys. This study now allows for a further acceleration of the identification of candidate species, by comparing newly obtained sequences with our database of reliably vouchered and often topotypical sequences. DNA barcoding may thereby play an eminent role and provide an effective and cost-efficient tool to help understanding the diversity of reptiles of Madagascar, although the Linnean shortfall remains. In fact, a preliminary and often molecular-only definition of a candidate species still requires a time-consuming species delimitation analysis and subsequently a formal scientific description. For future surveys of biotic diversity in Madagascar and elsewhere, we strongly recommend the collection of tissue samples for molecular analysis of all collected specimens. Furthermore, we suggest to use DNA barcoding for a large-scale screening of genetic diversity especially in groups such as the Madagascan reptiles and amphibians where large reference sequence databases now exist ([8], [88] this study).

Two major reasons might have positively influenced our barcoding analyses. First, our comprehensive sampling includes more than 250 nominal species. Second, the long duration of faunal survey work in Madagascar is likely responsible for removing many taxonomic artefacts. Such artefacts can lead to strong and hardly reconcilable inconsistencies between barcoding data and non-molecular data and thereby strongly reduce the utility and resolution of DNA barcoding.

The use of DNA barcodes has significant applications for conservation. Numerous species of reptiles from Madagascar are highly priced in the pet trade and exported from Madagascar in large numbers [89], [90]. Many of these species are listed in the appendices of the Convention on the International Trade of Endangered Species (CITES) and, thus, their commerce needs to be internationally monitored. At present, in Madagascar this applies to all chameleons, all tortoises and most turtles, all geckos of the genera *Uroplatus* and *Phelsuma*, Madagascan boas, and the Nile crocodile, a total of 140 species. Other species are subjected to export quotas from Madagascar or to recommendations to all CITES parties to suspend imports or to import bans in some countries (such as most species of chameleons in the European Union). Between 1985 and 2001, a total of 193,768 chameleons were legally exported from Madagascar and many instances of illegal trade have been recorded [89]. A reliable identification of all life stages of Madagascan reptiles is therefore a high priority in order to set up a sustainable trade system. Identification is not trivial given the difficulties in diagnosing juvenile and female chameleons. For example, the high level of morphological similarity among juveniles often confounds the identification of species, and even genera of chameleons. Also, many species of *Phelsuma* and *Uroplatus* are very difficult to tell morphologically apart even as adults. Recent advances in obtaining reliable DNA sequences from oral or cloacal swabbing [91]–[93] allows almost non-invasive sampling of reptiles thereby accounting for animal welfare. The COI database provides barcodes for about 110 of the 140 Madagascan reptiles included in CITES, and thereby provides a solid basis for future controls of the trade via molecular identification methods.

## Supporting Information

Figure S1 **Graph showing transitions and transversions plotted against K2p divergence.**
(PPT)

Figure S2 **Neighbor-joining tree based on COI sequences of Madagascan reptiles including specimen data.**
(PDF)

Figure S3 **Bayesian tree based on COI sequences of Madagascan reptiles.**
(PDF)

Figure S4 **Maximum likelihood tree based on COI sequences of Madagascan reptiles.**
(PDF)

Table S1 **Data of specimens and samples used in the study.**
(XLS)

Table S2 **Comparison of support values (bootstrap values, NJ analyses) for taxonomically relevant groups in the original data set and in simulations with subsampling.**
(DOC)
