# Supplementary figures and images for: First Large-Scale DNA Barcoding Assessment of Reptiles in the Biodiversity Hotspot of Madagascar, Based on Newly Designed COI Primers
Source: PLoS One. 2012 Mar 30;7(3):e34506. doi: 10.1371/journal.pone.0034506 (PMC3316696; doi:10.1371/journal.pone.0034506)

## Slide 1
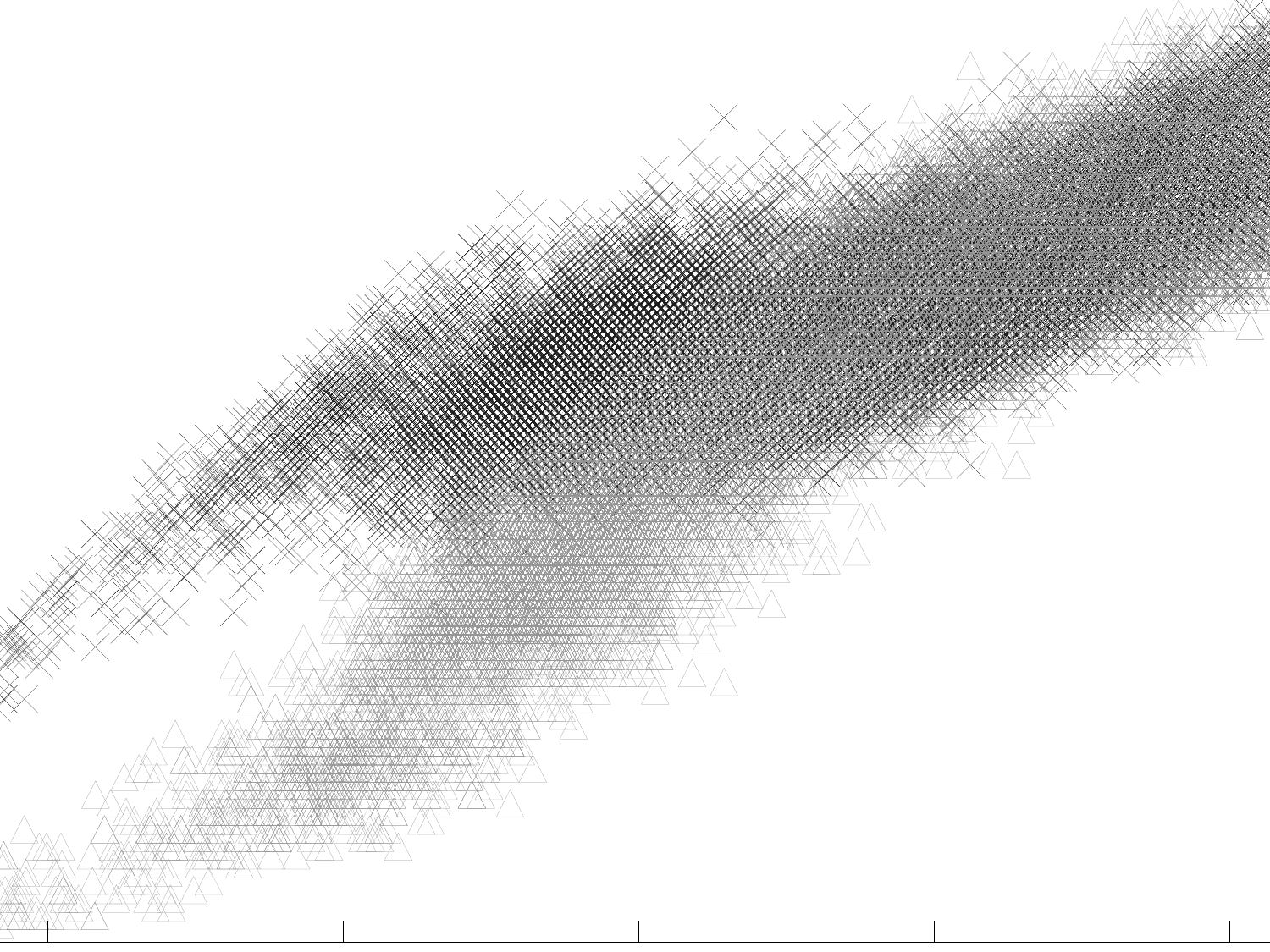

Supplement: Figure S1 — Graph showing transitions and transversions plotted against K2p divergence. (PPT) [file pone.0034506.s001.ppt]

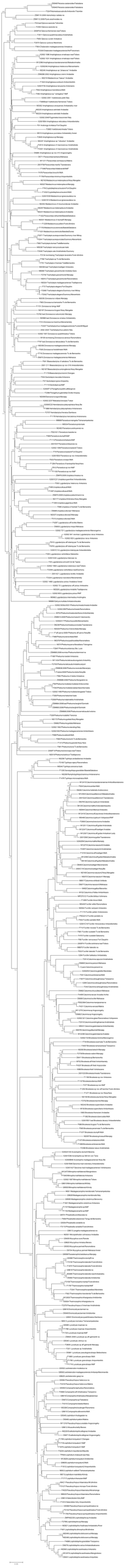

Supplement: Figure S2 — Neighbor-joining tree based on COI sequences of Madagascan reptiles including specimen data. (PDF) [file pone.0034506.s002.pdf]

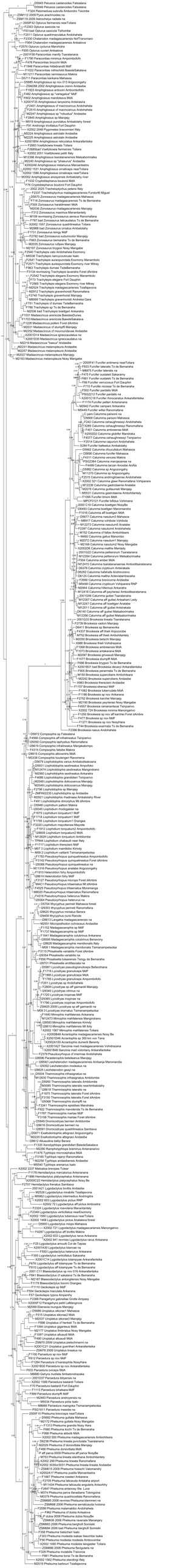

Supplement: Figure S3 — Bayesian tree based on COI sequences of Madagascan reptiles. (PDF) [file pone.0034506.s003.pdf]

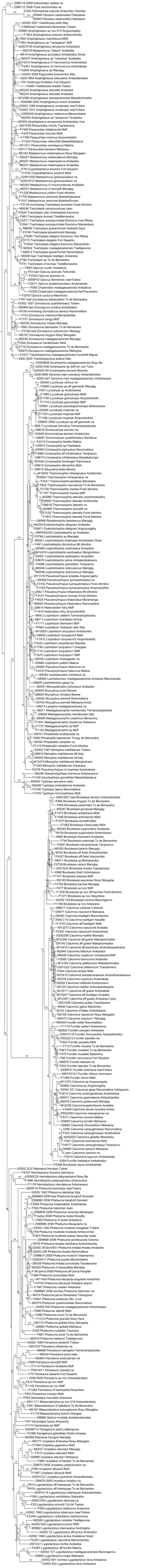

Supplement: Figure S4 — Maximum likelihood tree based on COI sequences of Madagascan reptiles. (PDF) [file pone.0034506.s004.pdf]
