# Supplementary material for: First Large-Scale DNA Barcoding Assessment of Reptiles in the Biodiversity Hotspot of Madagascar, Based on Newly Designed COI Primers
Source: PLoS One. 2012 Mar 30;7(3):e34506. doi: 10.1371/journal.pone.0034506 (PMC3316696; doi:10.1371/journal.pone.0034506)
Supplement: Table S2 — Comparison of support values (bootstrap values, NJ analyses) for taxonomically relevant groups in the original data set and in simulations with subsampling. (DOC) [file pone.0034506.s006.doc]

**Table S2.** Comparison of support values (bootstrap values, NJ analyses) for taxonomically relevant groups in the original data set and in simulations with subsampling.

| Family | Bootstrap value in the original data set | Range of support values in subsampling | Genus^1^ | Number of species | Support value in the original data set | Range of support values in subsampling |
| --- | --- | --- | --- | --- | --- | --- |
| Boidae | ca. 100 | 80-100 | *Acrantophis*  *Sanzinia* | 3  1 | 100  100 | 100  100 |
| Lamprophiidae | 0 | 0-100 | *Compsophis*  *Dromicodryas*  *Exallodontophis*  *Ithycyphus*  *Leioheterodon*  *Liophidium*  *Liopholidophis*  *Lycodryas*  *Madagascarophis*  *Phisalixella*  *Pseudoxyrhopus*  *Thamnosophis* | 7  2  1  3  3  9  7  7  3  3  6  6 | ca. 90  100  100  100  0  0  ca. 30  0  100  ca. 90  0  100 | 60-100  100  100  100  0-100  0-100  0-100  0-100  100  0-100  0-100  100 |
| Typhlopidae | 100 | 100 | *Typhlops* | 4 | 0 | 0-100 |
| Iguanidae | ca. 100 | 60-100 | *Chalarodon*  *Oplurus* | 1  5 | ca. 70  ca. 40 | 60-100  0-100 |
| Gekkonidae | ca. 10 | 0-100 | *Blaesodactylus*  *Geckolepis*  *Hemidactylus*  *Lygodactylus*  *Paragehyra*  *Paroedura*  *Phelsuma*  *Uroplatus* | 4  3  3  17  2  11  31  10 | ca. 20  ca. 0  0  0  ca. 30  10  ca. 60  0 | 0-100  0-80  0-100  0-100  0-90  0-100  0-100  0-100 |
| Gerrhosauridae | ca. 90 | 30-100 | *Tracheloptychus*  *Zonosaurus* | 2  12 | ca. 40  0 | 0-60  0-100 |
| Scincidae | 0 | 0-100 | *Amphiglossus*  *Cryptoblepharus*  *Madascincus*  *Paracontias*  *Trachylepis*  *Voeltzkowia* | 17  1  7  7  12  3 | 0  100  0  0  ca. 20  0 | 0-100  100  0-100  0-100  0-100  0-100 |
| Chamaeleonidae | 100 | 80-100 | *Brookesia*  *Calumma*  *Furcifer* | 27  37  13 | ca. 60  0  0 | 0-100  0-100  0-100 |
| Pelomedusidae | 100 | 100 | *Pelusios* | 1 | 100 | 100 |
| Psammophiidae | 100 | 100 | *Mimophis* | 1 | 100 | 100 |
| Testudinidae | 100 | 100 | *-* | - | - | - |

^1^ Genera represented by a single sequence in the data set were excluded from the analysis
